# Supplementary material for: Directing polymorph specific calcium carbonate formation with de novo protein templates
Source: Nat Commun. 2023 Dec 14;14:8191. doi: 10.1038/s41467-023-43608-1 (PMC10721895; doi:10.1038/s41467-023-43608-1)
Supplement: Supplementary file 1 — Supplementary Information [file 41467_2023_43608_MOESM1_ESM.pdf]

Supplementary Information

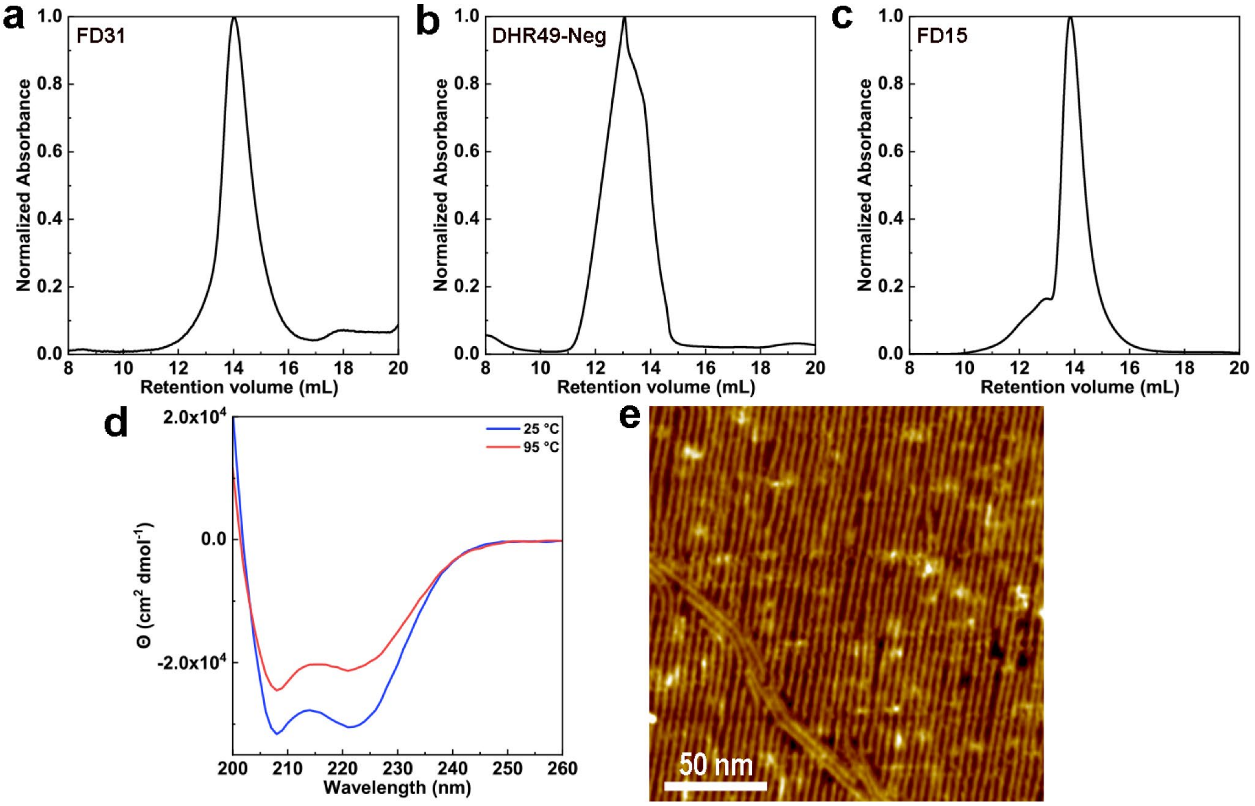

Supplementary Fig. 1. Biophysical characterization of selected proteins. Normalized ultraviolet absorbance (A230) of a size exclusion chromatography trace for (a) FD31, (b) DHR49-Neg, (c) FD15. (d) Circular dichroism scans from at 25 °C (blue) and 95 °C (red) for DHR49-Neg. (e) AFM image of DHR49-Neg assembly on mica in 3 M KCl solvent showing fiber-like behavior driven by end-to-end oligomerization.

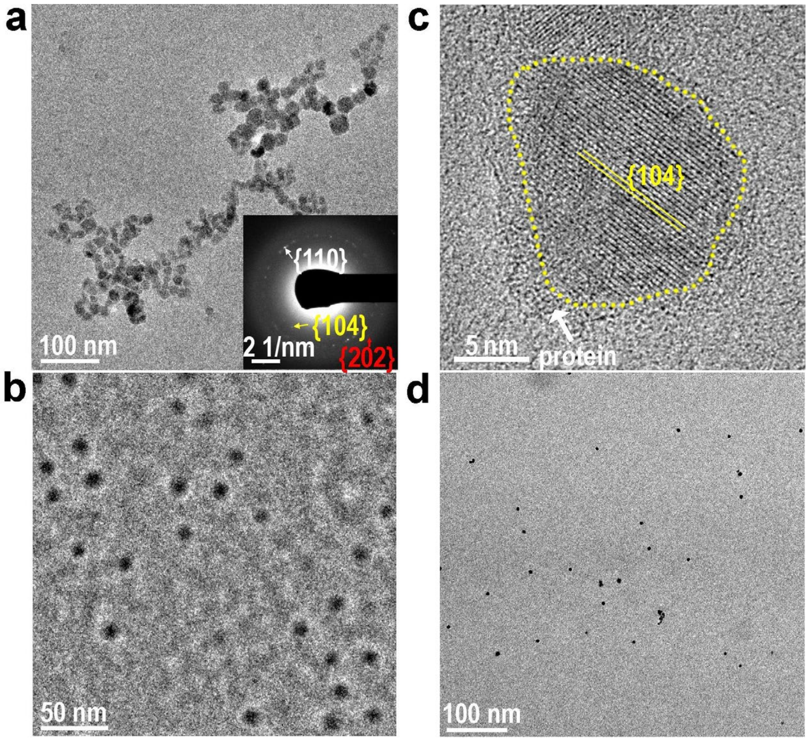

Supplementary Fig. 2. TEM images of calcite nanocrystals in the presence of 1.08 µM FD31. (a) TEM and corresponding SAED (inset) images confirm the nanocrystals are calcite. (b) Cryo-TEM image of $CaCO_3$ particles at 5 min. (c) HR-TEM image of calcite nanocrystals. The amorphous objectives with low contrast are suggestive of a shell around the nanocrystals, which could be absorbed protein (highlighted by white arrow). The yellow dotted line marks the calcite crystals. (d) LP-TEM image shows ≈ 5 nm $CaCO_3$ nanoparticles formed in the mineralization solution.

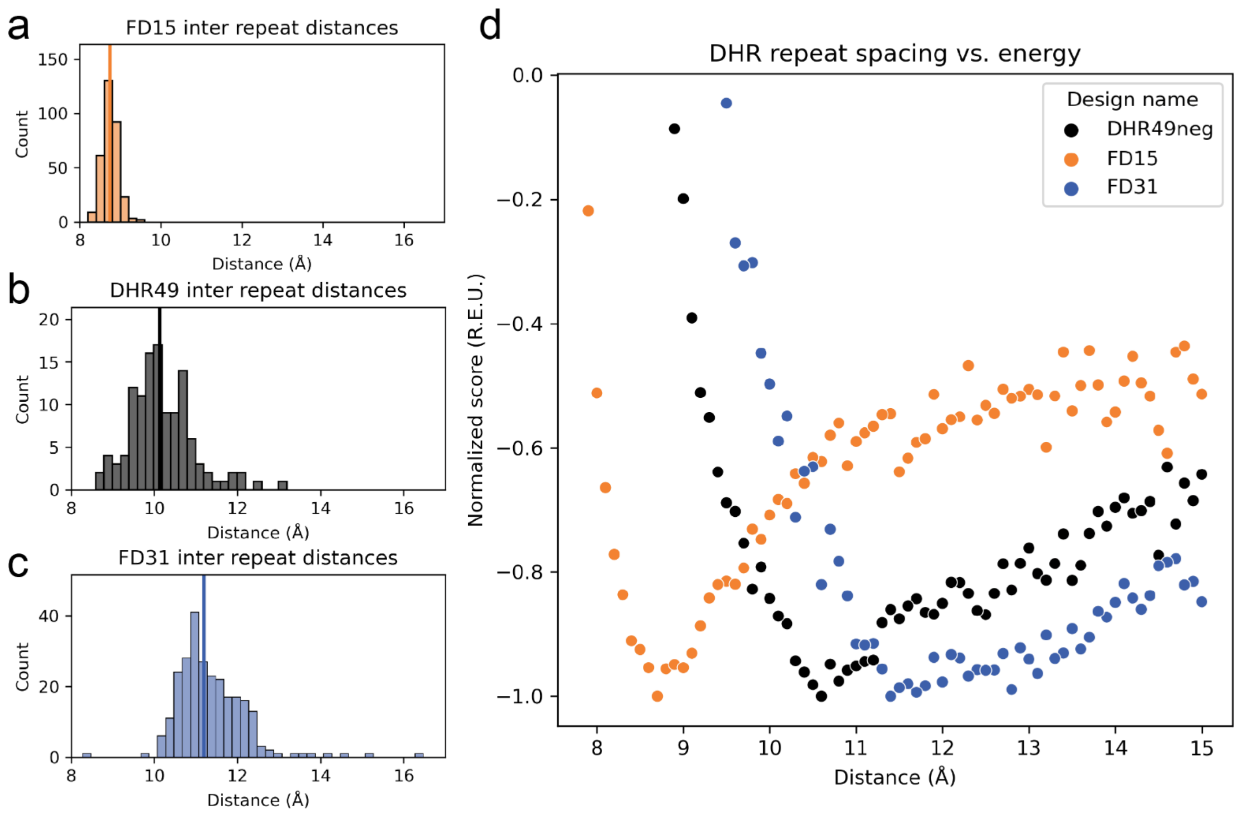

Supplementary Fig. 3. Inter-repeat distances in designed helical repeat proteins. Distribution of distances between α-carbon atoms in adjacent repeats within (a) FD15 crystal structure, (b) the DHR49-Neg crystal structure (pdb id: 5CWJ) that DHR49-Neg was derived from, and (c) the AlphaFold model of FD31. Lines show median values for each protein (FD15 = 8.7 Å; DHR49-Neg = 10.1 Å; FD31 = 11.2 Å). (d) Normalized predicted protein energy in Rosetta energy units (R.E.U.) vs. repeat-repeat distance (Å) for the lowest energy DHR models constrained to flat conformations with a specified repeat-repeat distance. The predicted energetic minima for DHR49-Neg is -510 R.E.U. at 10.6 Å, for FD15 is -919 R.E.U. at 8.7 Å, and for FD31 is -830 R.E.U. at 11.4 Å.

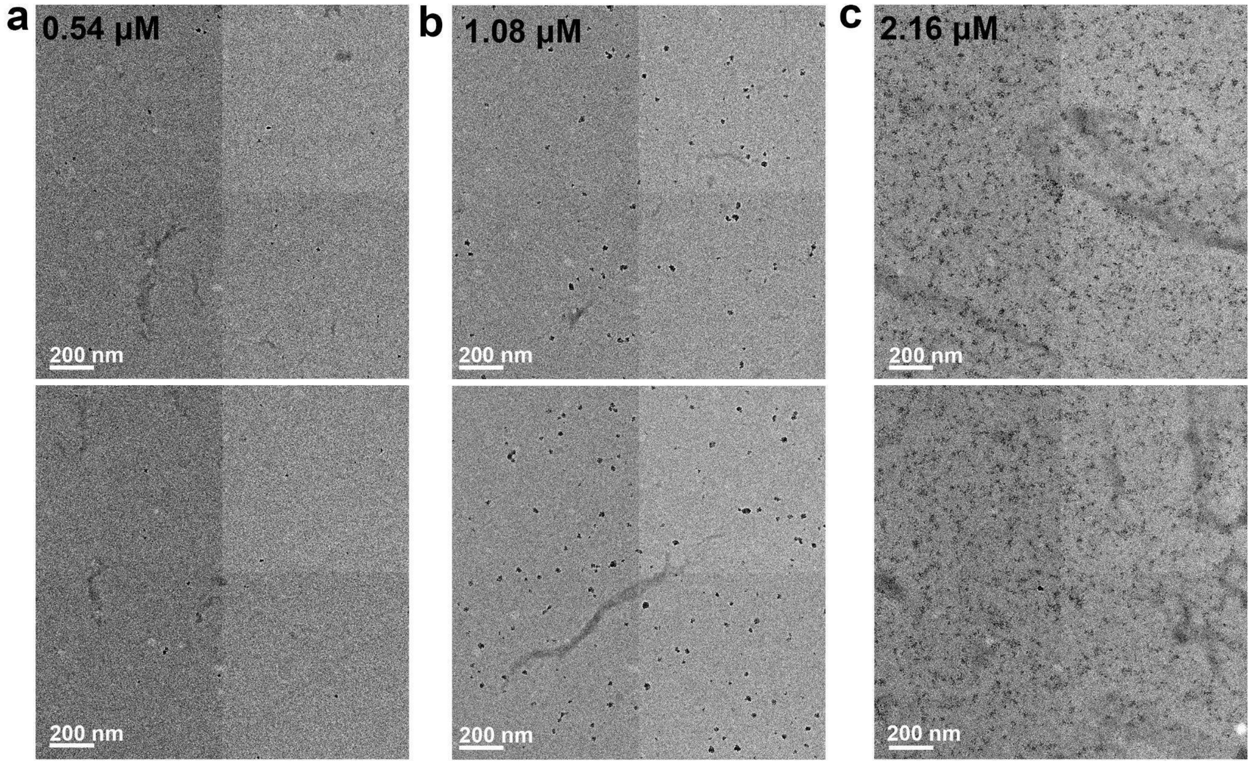

Supplementary Fig. 4. Effects of protein concentration on nucleation. FD31 concentration effects on $CaCO_3$ nucleation in supersaturated solutions containing 5 mM $CaCl_2$ and 5 mM $NaHCO_3$. FD31 was incubated with 10 mM $CaCl_2$ for 5 minutes prior to addition of an equal volume of 10 mM $NaHCO_3$. TEM samples were prepared 10-20 minutes after addition of $NaHCO_3$. (a-c) Representative TEM images show the resulting $CaCO_3$ particles on copper grids in the presence of 0.54, 1.08, 2.16 μM FD31 proteins. Every experiment was repeated 3 times with similar results.

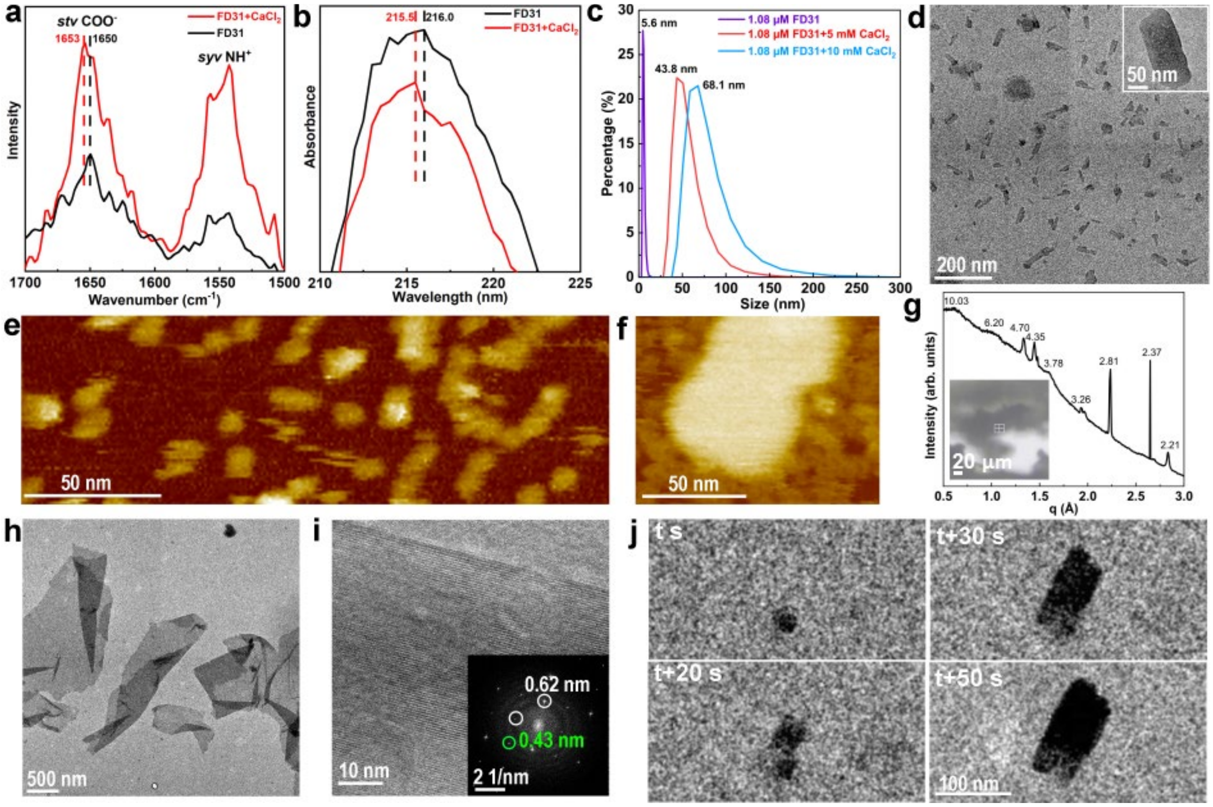

Supplementary Fig. 5. Structural characterization of the FD31-Ca complex. (a) Liquid-phase ATR-FTIR spectra and (b) UV-vis spectra of pure FD31 (black) and FD31 plus $CaCl_2$ (red) solutions. The peak shift shows the coordination of Ca with carboxylate groups on the protein. (c) DLS size measurements of FD31 with different $CaCl_2$ concentrations (0, 5, and 10 mM). (d) LP-TEM image showing the sheet-like assemblies. Inset shows individual assembly. (e-f) AFM images showing the rod-like individual FD31 molecules and FD31-Ca complex on mica with $CaCl_2$. Panel e reveals a rod-like shape of the FD31 protein molecules dispersed in MOPS buffer solution with a size of ≈ 8 nm by 5 nm by 2 nm (Length by width by height), which is consistent with their simulated "Flattened" Rosetta structure model (≈ 7.8 nm by 4.5 nm by 2.5 nm). (g) Beamline XRD of assembled FD31 protein-Ca^{2+} structure. The inset represents an optical microscopy image of the protein-Ca^{2+} assemblies. The marked area by white framework is the place exposed to the synchrotron X-ray beam. (h-i) Representative TEM image showing the sheet-like assemblies. The experiment was repeated 3 times with similar results. (j) Time-dependent LP-TEM images show the formation of the FD31-Ca complex.

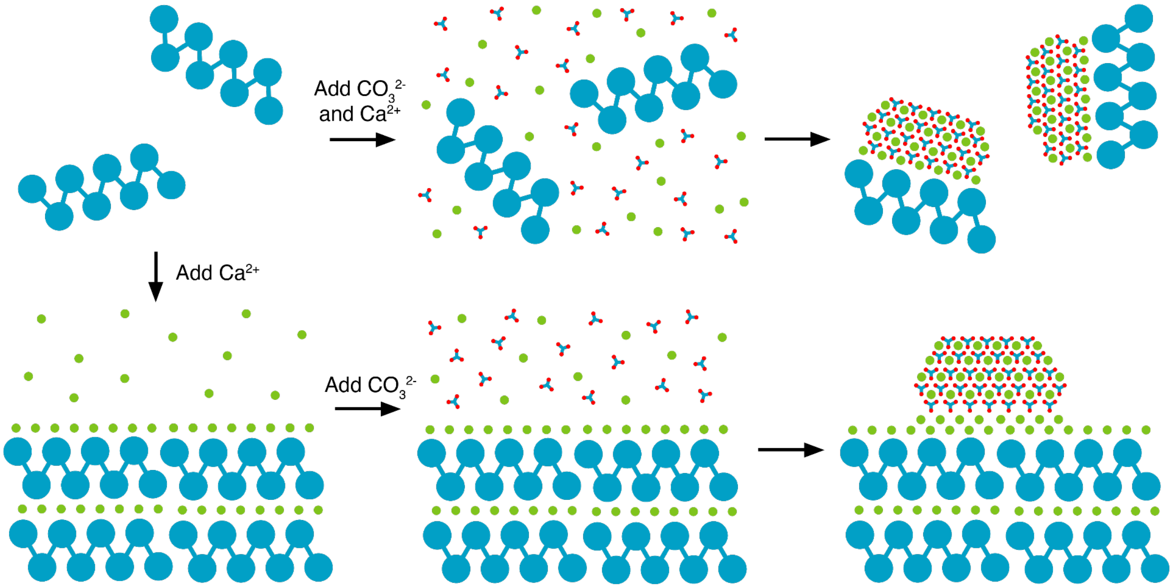

Supplementary Fig. 6. Proposed nucleation pathways for the FD31 system. (Top) when Ca^{2+} and CO_3^{2-} are mixed with protein simultaneously, nucleation of calcite is driven by protein monomers. (Bottom) when Ca^{2+} is added first, protein-Ca^{2+} assemblies form and serve as templates for calcite upon addition of CO_3^{2-}.

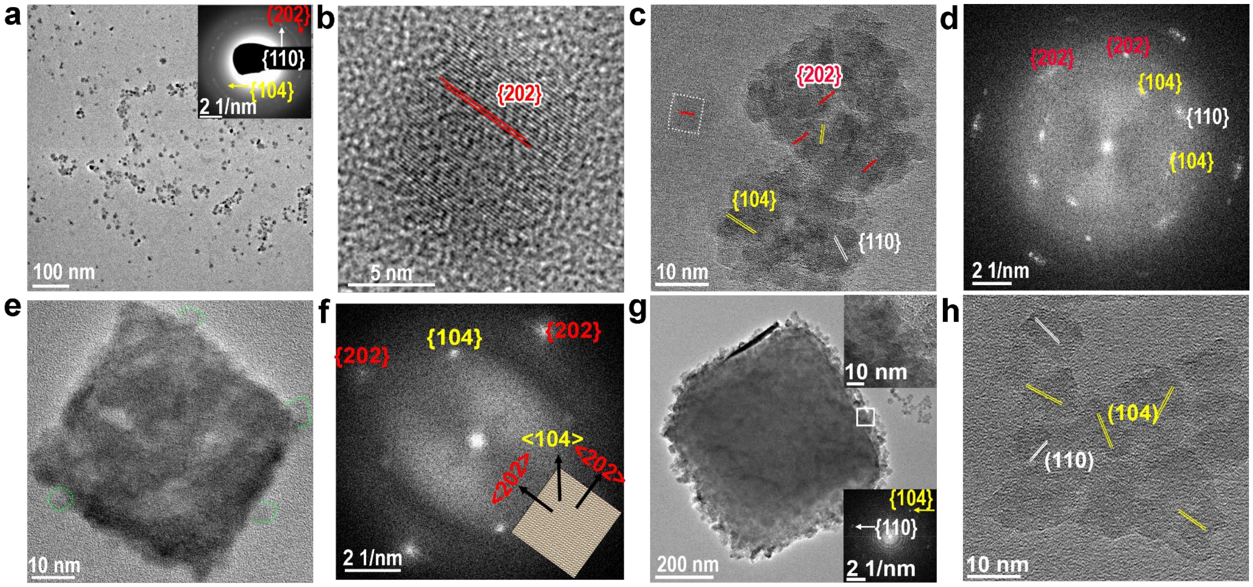

Supplementary Fig. 7. The $CaCO_3$ crystallization process in the presence of DHR49-Neg. (a) TEM and inserted SAED images show that the nanocrystals are calcite. (b) HR-TEM image shows an individual nanocrystal with {202} lattice direction. (c, d) HR-TEM and corresponding FFT images show aggregated calcite single crystals. (e, f) HR-TEM and corresponding FFT images show a cubic-like single crystal with exposed {202} facets. The inset in panel "f" shows the calcite shape and exposed facets. (g, h) Individual rhombohedral calcite with some nanocrystals on or around it. Inserted HR-TEM and FFT images in panel "g" to confirm that calcite nanocrystals are incorporated into the pre-existed calcite. The yellow and white lines represent {104} and {110} facets, respectively. The experiment was repeated 5 times with similar results.

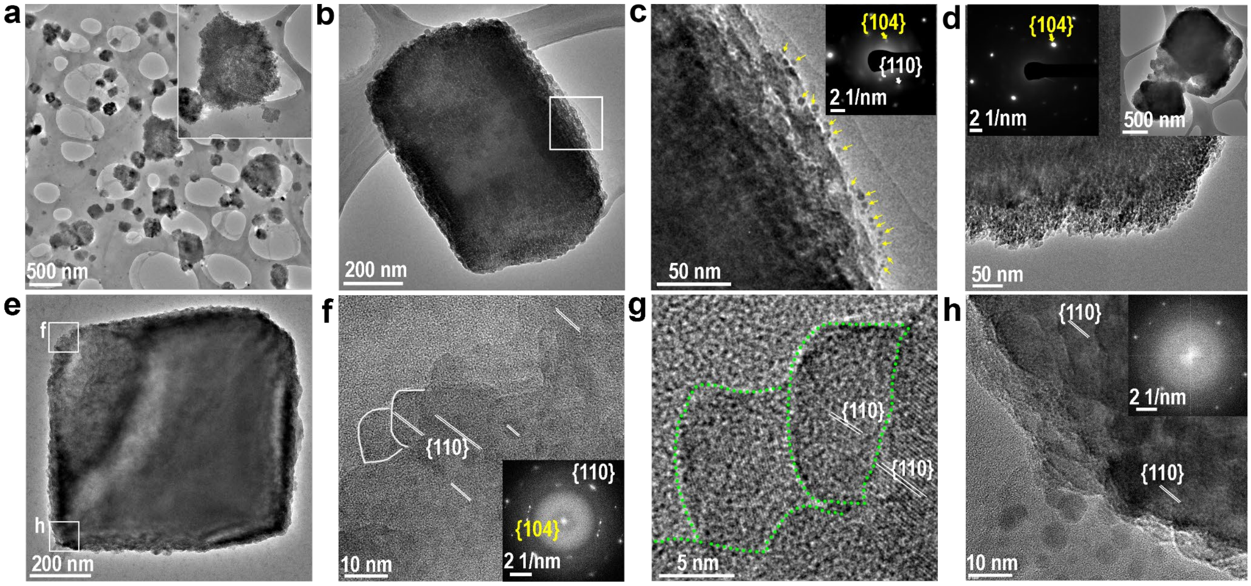

Supplementary Fig. 8. TEM images of calcite at 30 min in the presence of 1.08 μM FD31 protein. (a) Irregular calcite consisting of nanocrystals. Inset shows an individual calcite aggregate. (b, c) One calcite crystal is surrounded by some calcite nanoparticles. Inserted SAED image confirms the single crystalline nature. (d) Rhombohedral calcite crystals consist of smaller nanocrystals. (e) TEM image shows a rhombohedral calcite with a size of ≈ 800 nm. (f, g, h) Higher magnification views of the corners of the particle in (e). (f) HR-TEM image of the corner region showing the same {110} lattice orientation as in the crystal body. (g) HR-TEM image showing several single-crystal units on the surface. Green dotted lines highlight the calcite building units. (h) HR-TEM image showing numerous small nanocrystals located around the rhombohedral calcite. The experiment was repeated 5 times with similar results.

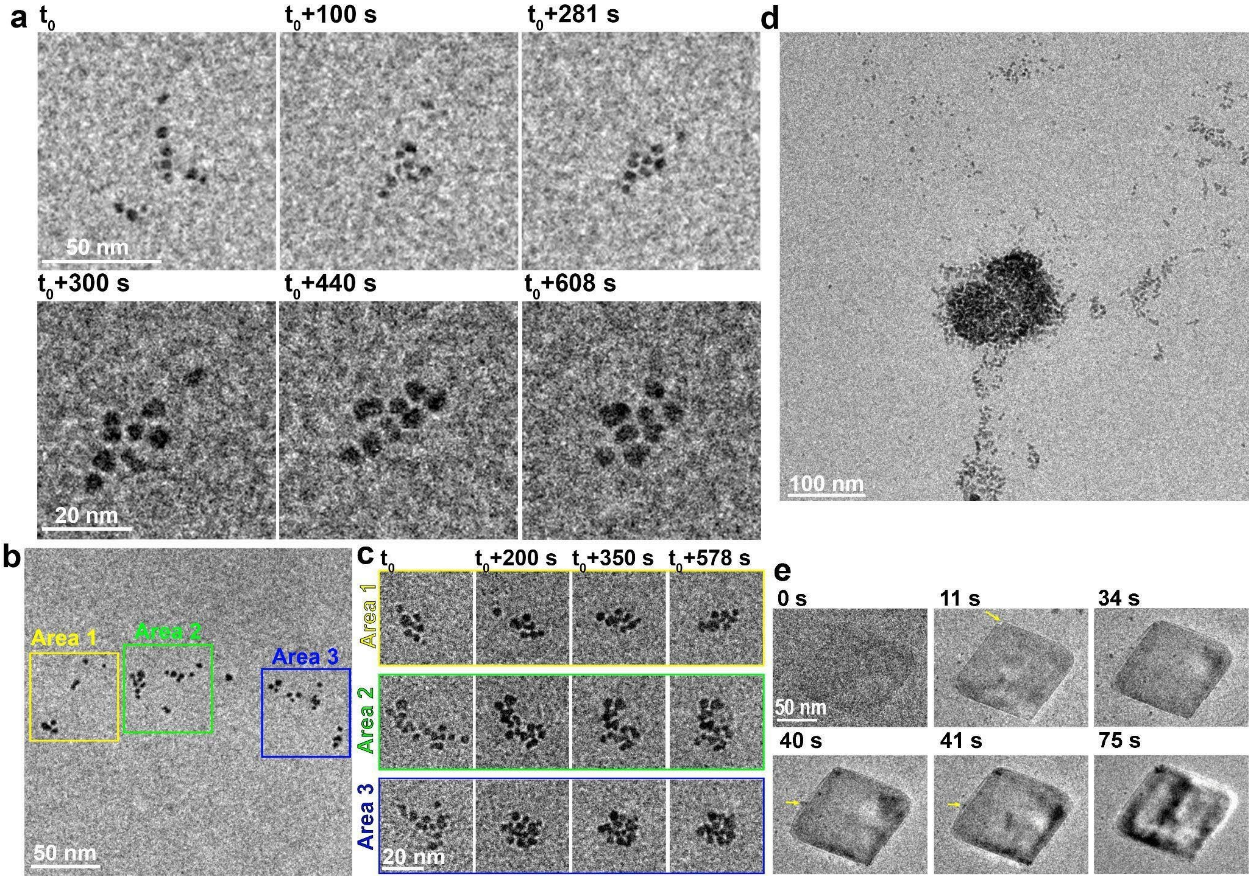

Supplementary Fig. 9. LP-TEM observations of the dynamic behavior of calcite nanoparticles grown in the liquid-cell in the presence of FD31. (a) Sequential TEM images showing the aggregation process of multiple particles. (b) LP-TEM image showing the initial distribution of particles. Yellow, green, and blue frames highlight the 3 selected areas. (c) Series of TEM images showing the formation of three aggregates. (d) LP-TEM image showing the aggregated particles. (e) Sequential TEM images show the attachment of particles into pre-existed rhombohedral calcite. Yellow arrows show the individual particles around rhombohedral calcite. The *in-situ* TEM experiment was repeated 3 times.

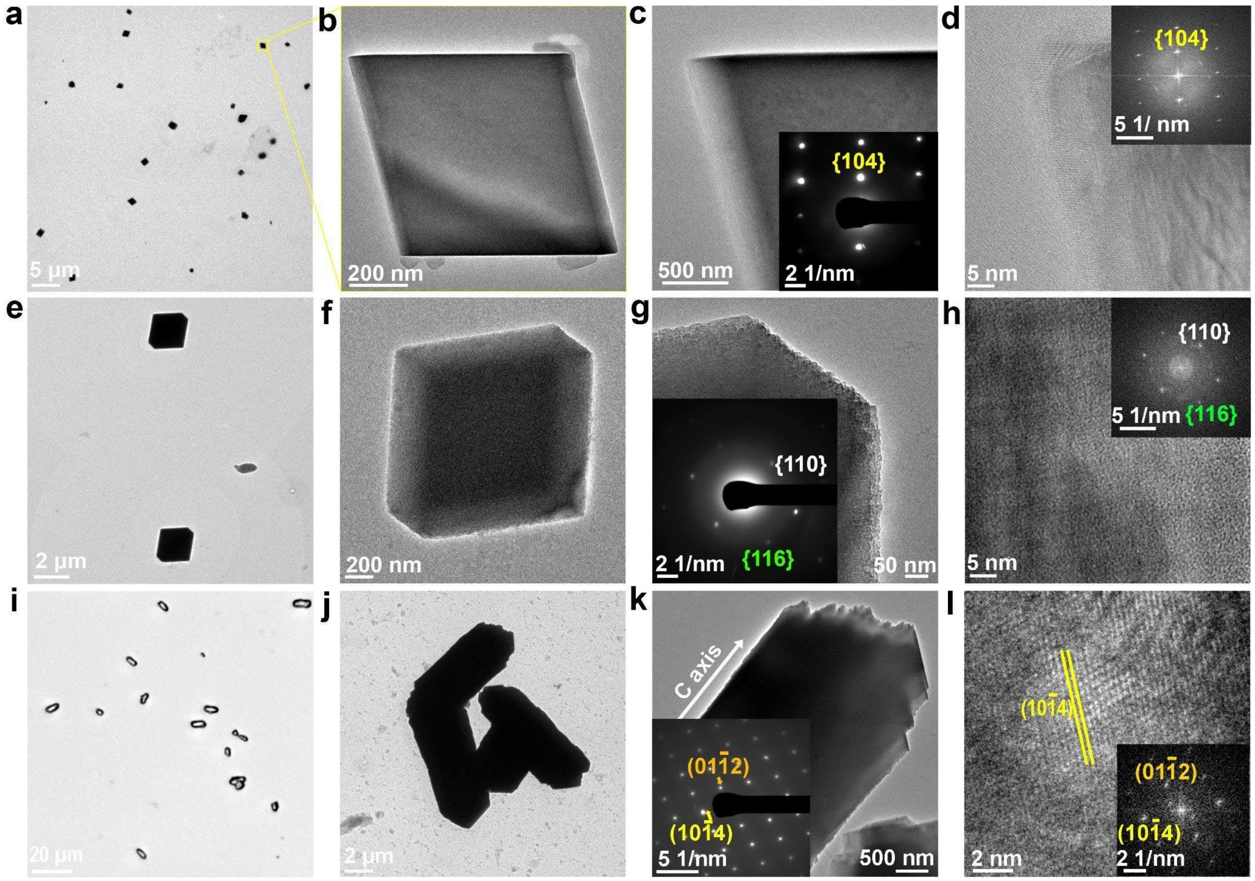

Supplementary Fig. 10. Effects of protein concentration on post nucleation growth. The resulting $CaCO_3$ morphology after 8 hours without additives or with proteins of different concentrations in supersaturated solutions containing 5 mM $CaCl_2$ and 5 mM $NaHCO_3$. (a-d) TEM and SAED images show the formation of perfect rhombohedral calcite without the involvement of proteins. (e-h) TEM and SAED images show the formation of rough rhombohedral calcite with the addition of 1.08 µM FD31 proteins. (i) Optical microscopy image (k), TEM and SAED images show the formation of rod-like calcite with the addition of 4.32 µM FD31 protein. The experiment was repeated 3 times with similar results.

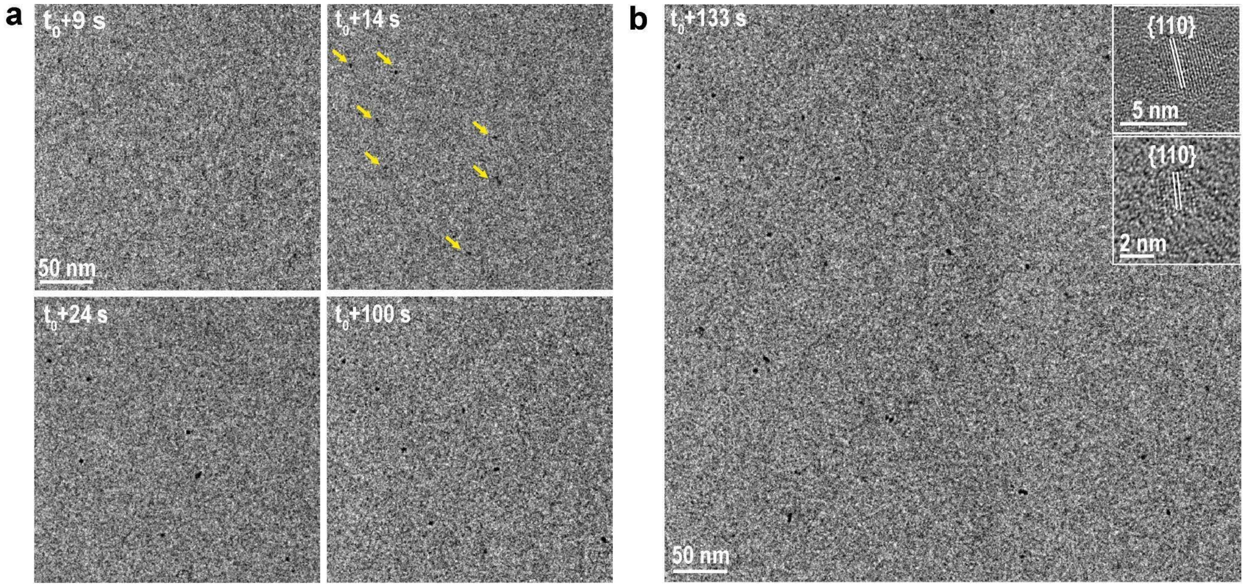

Supplementary Fig. 11. *In situ* LP-TEM observation of $CaCO_3$ crystallization process in the presence of FD31-Rep9 protein. (a) Sequential TEM images showing the nucleation of $CaCO_3$ nanoparticles. The yellow arrows highlight the newly formed particles. (b) TEM image showing multiple $CaCO_3$ nanoparticles in the liquid cell. Inserted HR-TEM image confirms the nanocrystals are calcite.

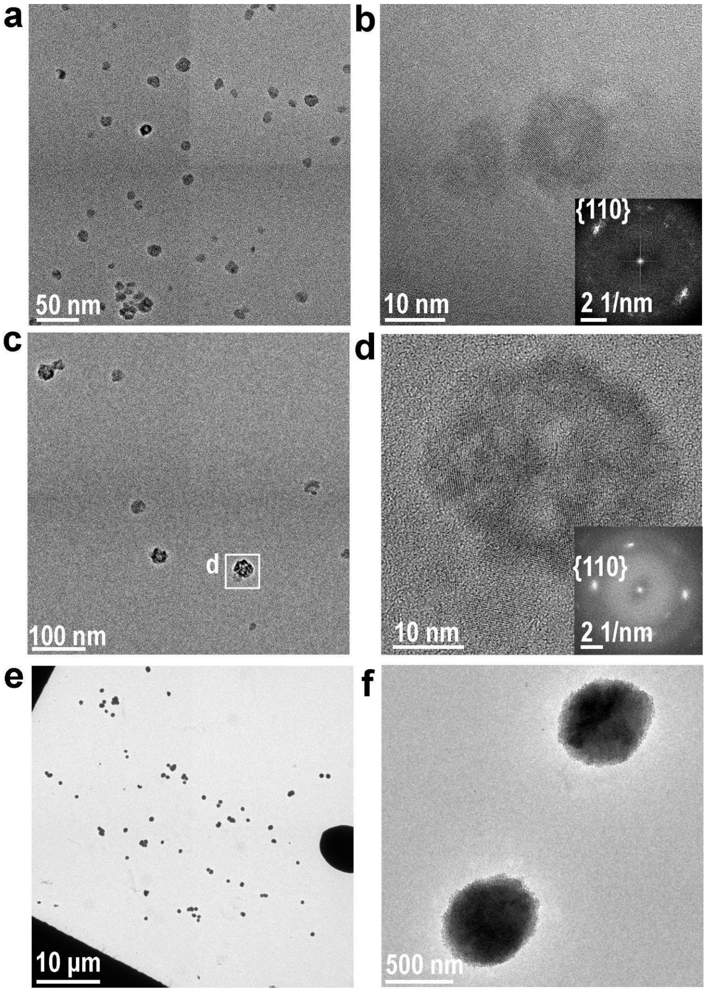

Supplementary Fig. 12. $CaCO_3$ formed in the presence of different FD31-mutants. (a-b) TEM and SAED images show the formation of calcite-dominant nanocrystals in the presence of FD31-Gln-Checker. (c-d) TEM and FFT images show some calcite nanocrystals in the presence of FD31-Asp. (e-f) TEM and SAED images show the formation of a dominant vaterite phase in the presence of FD31-Lys-Checker. The experiment was repeated 3 times with similar results.

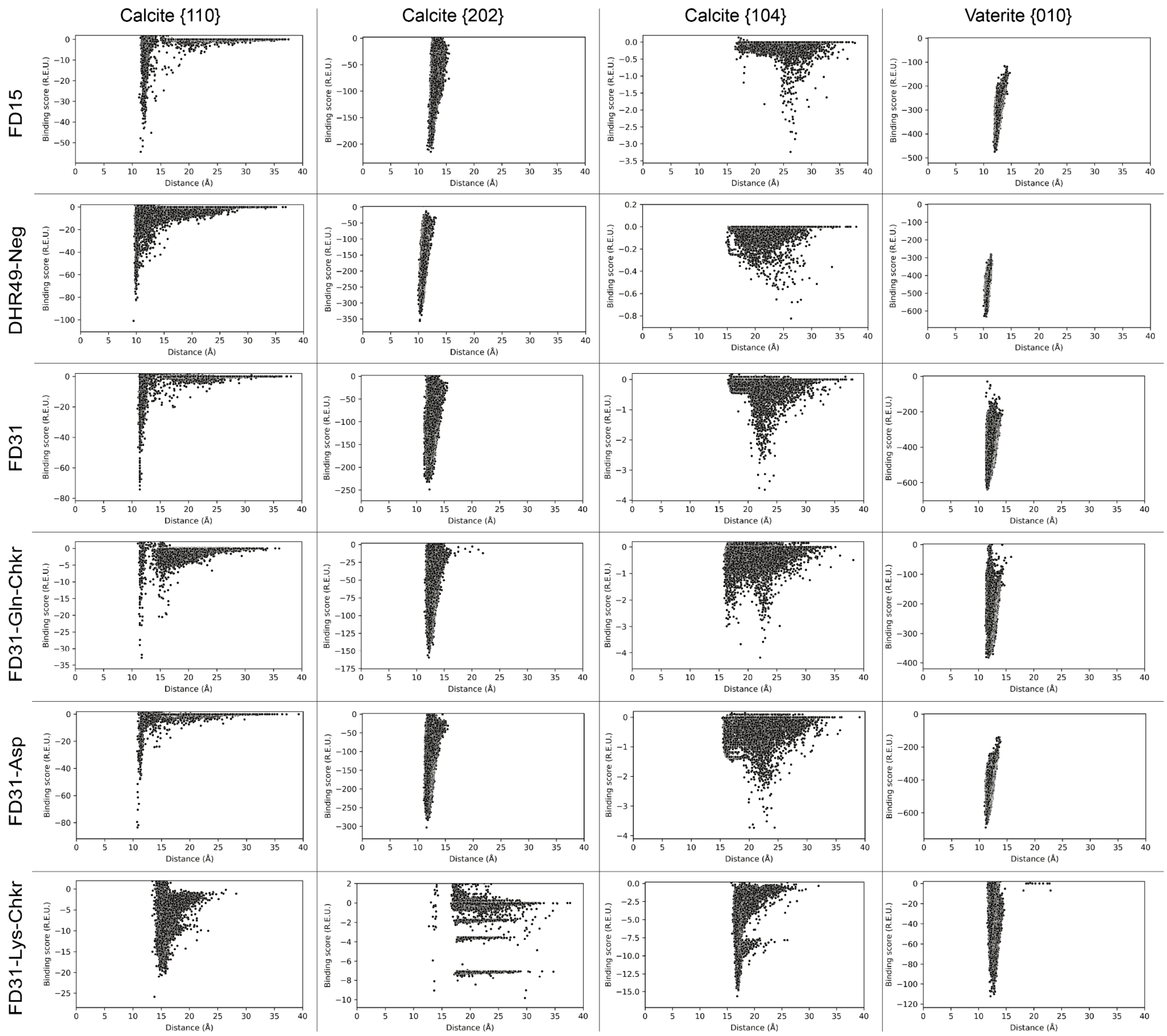

Supplementary Fig. 13. Rosetta binding score vs. distance of proteins to $CaCO_3$ surfaces. Binding score vs. distance from the protein's center of mass to the surface in all Rosetta docking trajectories of DHRs on four $CaCO_3$ surfaces. To allow for the possibility of small conformational changes in the proteins upon adsorption to the mineral surface, idealized models of each DHR with a range of inter-repeat spacings at 0.1 Å increments were docked on the surfaces (8.0 Å to 9.7 Å for FD15, 9.6 Å to 11.6 Å for DHR49-Neg, and 10.4 Å to 12.4 Å for FD31).

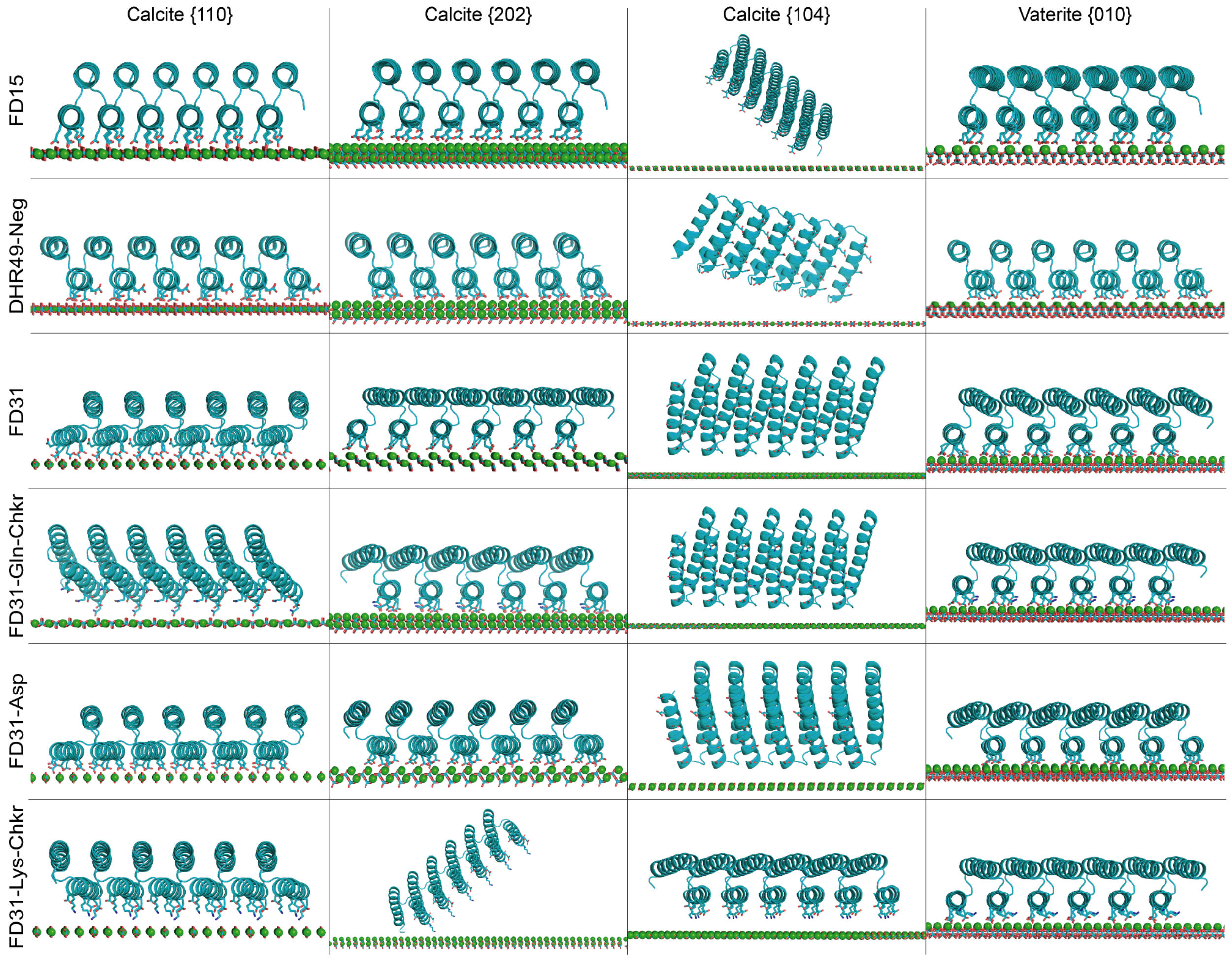

Supplementary Fig. 14. Low energy Rosetta models of DHRs docked on $CaCO_3$. These models illustrate how the repeat proteins may geometrically match the $CaCO_3$ facets.

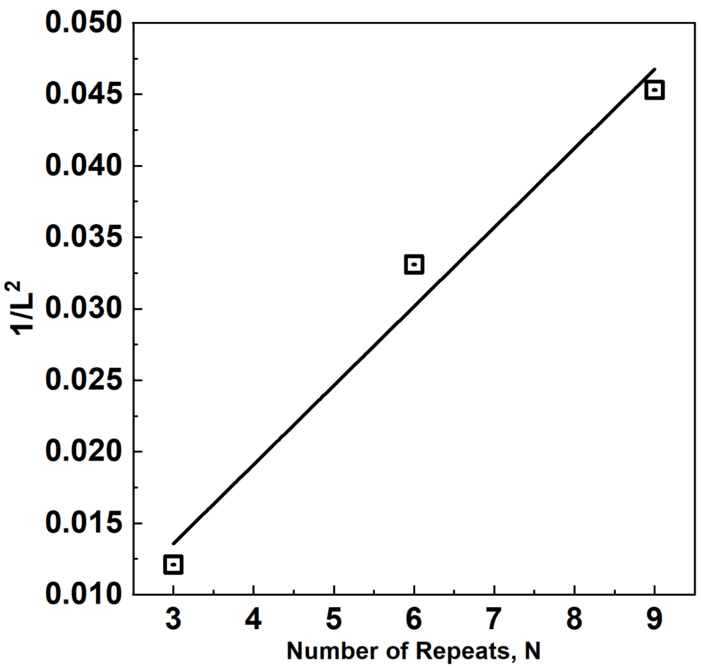

Supplementary Fig. 15. Dependence of nanocrystal size on length of DHR protein template, where *L* is the average nanocrystal diameter and N is the number of repeats in the protein. The nanocrystal surface area is proportional to L^2 and the protein surface area is proportional to *N*.

Supplementary Table 1: Sequences of tested proteins. *Indicates well expressing sequences (> 20 mg/L of culture, after purification). **Indicates sequences that were further characterized and tested in nucleation trials. ***Indicates variants of FD31 that were tested for nucleation activity. Underlined sequences were removed during purification by cleavage with TEV protease.

Name	Sequence
FD15**	MGSSHHHHHHSSGLVPRGSHMENLYFQGSWSGGSGGEAADEARRAIEAALEEARAAADE<br>ARSDSTGETVKKAVDKAEKAAEDAFREIKQAVNQAEKQGASEAAFEAFAAIAAAAAEAA<br>AAAFEAFSDSTGETVAEAVAKALKAAMEAFAEIAKAVAQAAKQGASEAAFEAFAAIAAA<br>AAEAAAAAFEAFSDSTGETVAEAVAKALKAAMEAFAEIAKAVAQAAKQGASEAAFEAFA<br>AIAAAAAEAAAAAFEAFSDSTGETVAEAVAKALKAAMEAFAEIAKAVAQAAKQGASEAA<br>FEAFAAIAAAAAEAAAAAFEAFSDSTGETVAEAVAKALKAAMEAFAEIAKAVAQAAKQG<br>ASEEAFEKFAAIAAEAAEAAAAAFERFSDSTGETEAEKVAKELKQLMEEFAERAKSVAEQ<br>AKNGAS
DHR49-Neg**	MDSKVLEEAIRVIAEIAKESGSEDAAESAIDAVADIADEAQDSKVLEEAIRVIAEIAKESGSE<br>DAAESAIDAVADIADEAQDSKVLEEAIRVIAEIAKESGSEDAAESAIDAVADIADEAQDSKV<br>LEEAIRVIAEIAKESGSEDAAESAIDAVADIADEAQDSKVLEEAIRVIAEIAKESGSEDAAESA<br>IDAVADIADEAQDSKVLEEAIRVIAEIAKESGSEDAAESAIDAVADIADEAQGW
FD31**	MGSSHHHHHHSSGLVPRGSHMENLYFQGSWSGGSGGPEEALEEVEERIEELESALESNPT<br>NEEELREILKKILEIFEELFREAKARNDTELLLEAVEAVIELLETLLELNPTNEELLREILKIIL<br>RIFELLFELAKKQNDTELLLEAVEAVIELLETLLELNPTNEELLREILKIILRIFELLFELAKKQ<br>NDTELLLEAVEAVIELLETLLELNPTNEELLREILKIILRIFELLFELAKKQNDTELLLEAVEA<br>VIELLETLLELNPTNEELLREILKIILRIFELLFELAKKQNDTELLSEAKEAVAELLETLAELN<br>PTNQELKEEIKKIQERIAELEKELAEKQNA
FD31-Rep3***	MGSSHHHHHHSSGLVPRGSHMENLYFQGSWSGGSGGPEEALEEVEERIEELESALESNPTN<br>EEELREILKKILEIFEELFREAKARNDTELLLEAVEAVIELLETLLELNPTNEELLREILKIILRI<br>FELLFELAKKQNDTELLSEAKEAVAELLETLAELNPTNQELKEEIKKIQERIAELEKELAEKQ<br>NA
FD31-Rep9***	MGSSHHHHHHSSGLVPRGSHMENLYFQGSWSGGSGGPEEASEEVEERSEELESALESNPTN<br>EEELREILKKIQEIFEELFREAKARNDTELLSEAVEAVSELLETLLENNPTNEELLREILKIIQR<br>IFELLFELAKKQNDTELLSEAVEAVSELLETLLENNPTNEELLREILKIIQRIFELLFELAKKQ<br>NDTELLSEAVEAVSELLETLLENNPTNEELLREILKIIQRIFELLFELAKKQNDTELLSEAVEA<br>VSELLETLLENNPTNEELLREILKIIQRIFELLFELAKKQNDTELLSEAVEAVSELLETLLENN<br>PTNEELLREILKIIQRIFELLFELAKKQNDTELLSEAVEAVSELLETLLENNPTNEELLREILKII<br>QRIFELLFELAKKQNDTELLSEAVEAVSELLETLLENNPTNEELLREILKIIQRIFELLFELAK<br>KQNDTELLSEAKEAVSELLETLAENNPTNQELKEEIKKIQERIAELEKELAEKQNA
FD31-Asp***	MGSSHHHHHHSSGLVPRGSHMENLYFQGSWSGGSGGPEEALDEVDERIDELESALDSNPT<br>NEEELREILKKILEIFEELFREAKARNDTELLLDAVDAVIDLLETLLDLNPTNEELLREILKIIL<br>RIFELLFELAKKQNDTELLLDAVDAVIDLLETLLDLNPTNEELLREILKIILRIFELLFELAKK<br>QNDTELLLDAVDAVIDLLETLLDLNPTNEELLREILKIILRIFELLFELAKKQNDTELLLDAV<br>DAAIDLLETLLDLNPTNEELLREILKIILRIFELLFELAKKQNDTELLSDAKDAVADLLETLA<br>DLNPTNQELKEEIKKIQERIAELEKELAEKQNA
FD31-Gln-	MGSSHHHHHHSSGLVPRGSHMENLYFQGSWSGGSGGPEEALQEVEERIQELESALQSNPT

Checker***	NEEELREILKKILEIFEELFREAKARNDTQLLLEAVQAVIELLQTLLELNPTNEELLREILKIILRIFELLFELAKKQNDTELLLQAVEAVIQLLETLLQLNPTNEELLREILKIILRIFELLFELAKKQNDTQLLLEAVQAVIELLQTLLELNPTNEELLREILKIILRIFELLFELAKKQNDTELLLQAVEAAIQLLETLLQLNPTNEELLREILKIILRIFELLFELAKKQNDTQLLSEAKQAVAELLQTLAELNPTNQELKEEIKKIQERIAELEKELAEKQNA
FD31-Lys-Checker***	MGSSHHHHHHSSGLVPRGSHMENLYFQGSWSGGSGGPEEALKEVEERIEELKSALESNPTNEEELREILKKILEIFEELFREAKARNDTKLLLEAVKAVIELLETLLKLNPTNEELLREILKIILRIFELLFELAKKQNDTELLLEAVEAVIKLLETLLELNPTNEELLREILKIILRIFELLFELAKKQNDTELLLKAVEAVIELLKTLLELNPTNEELLREILKIILRIFELLFELAKKQNDTKLLLEAVKAAIELLETLLKLNPTNEELLREILKIILRIFELLFELAKKQNDTELLSEAKEAVAKLLETLAELNPTNQELKEEIKKIQERIAELEKELAEKQNA
FD01	MGSSHHHHHHSSGLVPRGSHMENLYFQGSWSGGSGGPEELLREVRERIEELKSLLESNPTNEELLREILKKILEIFEELFREAKARNDVELLLRAVRLVIELLKTLLELNPTNEELLREILKIILRIFELLFELAKKLNDVELLLRAVRLVIELLKTLLELNPTNEELLREILKIILRIFELLFELAKKLNDVELLLRAVRLVIELLKTLLELNPTNEELLREILKIILRIFELLFELAKKLNDVELLLRAVRLVIELLKTLLELNPTNEELLREILKIILRIFELLFELAKKLNDTELLSRAKELVAELLRTLLELNPTNQELLEEIKKIQERIAELEKELLEKLNA
FD02	MGSSHHHHHHSSGLVPRGSHMENLYFQGSWSGGSGGPEELLAEVESRIAELESLLASNPTNEELLREILKKILEIFEELFREAKARNDVELLLAAVELVIALLETLLALNPTNEELLREILKIILRIFELLFELAKKLNDVELLLAAVELVIALLETLLALNPTNEELLREILKIILRIFELLFELAKKLNDVELLLAAVELVIALLETLLALNPTNEELLREILKIILRIFELLFELAKKLNDVELLLAAVELVIALLETLLALNPTNEELLREILKIILRIFELLFELAKKLNDTELLSAAKELVAELLETLLELNPTNQELLEEIKKIQERIAELEKELLEKLNA
FD03*	MGSSHHHHHHSSGLVPRGSHMENLYFQGSWSGGSGGPEELLREILKIILRIFELLFELAKKLNDVELLLAAVELVIALLETLLALNPTNEELLREILKIILRIFELLFELAKKLNDVELLLAAVELVIALLETLLALNPTNEELLREILKIILRIFELLFELAKKLNDVELLLAAVELVIALLETLLALNPTNEELLREILKIILRIFELLFELAKKLNDVELLLAAVELVIALLETLLALNP
FD04	MGSSHHHHHHSSGLVPRGSHMENLYFQGSWSGGSGGPELLLAAVEAVIAALETLLALNPTNEELLREILKIILRIFEALFELAKKLNDVELLLAAVELVIALLETLLALNPTNEELLREILKIILRIFELLFELAKKLNDVELLLAAVELVIALLETLLALNPTNEELLREILKIILRIFELLFELAKKLNDVELLAAAVELAAALLETLLALNPTNEELLREILKIIARIAELLAELAKKLN
FD05	MGSSHHHHHHSSGLVPRGSHMENLYFQGSWSGGSGGPAELLEEVESRIEELESLLASNPTNEELLREILKKILEIFEELFREAKARNDVALLLEAVELVIELLETLLALNPTNEELLREILKIILRIFELLFELAKKLNDVALLLEAVELVIELLETLLALNPTNEELLREILKIILRIFELLFELAKKLNDVALLLEAVELVIELLETLLALNPTNEELLREILKIILRIFELLFELAKKLNDVALLLEAVELVIELLETLLALNPTNEELLREILKIILRIFELLFELAKKLNDTALLSEAIELVAELLETLLALNPTNQELLEEIKKIQERIAELEKELLEKLNA
FD06	MGSSHHHHHHSSGLVPRGSHMENLYFQGSWSGGSGGPEELLREILKIILRIFELLFELAKKLNDVALLLEAVELVIELLETLLALNPTNEELLREILKIILRIFELLFELAKKLNDVALLLEAVELVIELLETLLALNPTNEELLREILKIILRIFELLFELAKKLNDVALLLEAVELVIELLETLLALNPTNEELLREILKIILRIFELLFELAKKLNDVALLLEAVELVIELLETLLALNP

FD07	MGSSHHHHHHSSGLVPRGSHMENLYFQGSWSGGSGGPALLLEAVEAVIEALETLLALNPTNEELLREILKIILRIFEALFELAKKLNDVALLLEAVELVIELLETLLALNPTNEELLREILKIILRIFELLFELAKKLNDVALLLEAVELVIELLETLLALNPTNEELLREILKIILRIFELLFELAKKLNDVALLAEAVELAAELLETLLALNPTNEELLREILKIIARIAELLAELAKKLN
FD08*	MGSSHHHHHHSSGLVPRGSHMENLYFQGSWSGGSGGPALLLEAVEAVIEALETLLALNPTNEELLREILKIILRIFEALFELAKKLNDVALLLEAVELVIELLETLLALNPTNEELLREILKIILRIFELLFELAKKLNDVALLLEAVELVIELLETLLALNPTNEELLREILKIILRIFELLFELAKKLNDVALLAEAVELAIELLETLLALNPTNEELLREIAKIIARIFELLAELAKKLN
FD09	MGSSHHHHHHSSGLVPRGSHMENLYFQGSWSGGSGGPEELLEEVESRIEELESLLESNPTNEELLREILKKILEIFEELFREAKARNDVELLLEAVELVIELLETLLELNPTNEELLREILKIILRIFELLFELAKKLNDVELLLEAVELVIELLETLLELNPTNEELLREILKIILRIFELLFELAKKLNDVELLLEAVELVIELLETLLELNPTNEELLREILKIILRIFELLFELAKKLNDVELLLEAVELVIELLETLLELNPTNEELLREILKIILRIFELLFELAKKLNDTELLSEAIELVAELLETLLELNPTNQELLEEIKKIQERIAELEKELLEKLNA
FD10	MGSSHHHHHHSSGLVPRGSHMENLYFQGSWSGGSGGPEELLAEVESRIAELESLLESNPTNEELLREILKKILEIFEELFREAKARNDVELLLAAVELVIALLETLLELNPTNEELLREILKIILRIFELLFELAKKLNDVELLLAAVELVIALLETLLELNPTNEELLREILKIILRIFELLFELAKKLNDVELLLAAVELVIALLETLLELNPTNEELLREILKIILRIFELLFELAKKLNDVELLLAAVELVIALLETLLELNPTNEELLREILKIILRIFELLFELAKKLNDTELLSAAIELVAALLETLLELNPTNQELLEEIKKIQERIAELEKELLEKLNA
FD11	MGSSHHHHHHSSGLVPRGSHMENLYFQGSWSGGSGGPDELLDEVDSRIDELDSLLDSNPTNEELLREILKKILEIFEELFREAKARNDVDLLLDAVDLVIDLLDTLLDLNPTNEELLREILKIILRIFELLFELAKKLNDVDLLLDAVDLVIDLLDTLLDLNPTNEELLREILKIILRIFELLFELAKKLNDVDLLLDAVDLVIDLLDTLLDLNPTNEELLREILKIILRIFELLFELAKKLNDVDLLLDAVDLVIDLLDTLLDLNPTNEELLREILKIILRIFELLFELAKKLNDTDLLSDAIDLVADLLDTLLDLNPTNQELLEEIKKIQERIAELEKELLEKLNA
FD12	MGSSHHHHHHSSGLVPRGSHMENLYFQGSWSGGSGGPDELLAEVDSRIAELDSLLDSNPTNEELLREILKKILEIFEELFREAKARNDVDLLLAAVDLVIALLDTLLDLNPTNEELLREILKIILRIFELLFELAKKLNDVDLLLAAVDLVIALLDTLLDLNPTNEELLREILKIILRIFELLFELAKKLNDVDLLLAAVDLVIALLDTLLDLNPTNEELLREILKIILRIFELLFELAKKLNDVDLLLAAVDLVIALLDTLLDLNPTNEELLREILKIILRIFELLFELAKKLNDTDLLSAAIDLVAALLDTLLDLNPTNQELLEEIKKIQERIAELEKELLEKLNA
FD13	MGSSHHHHHHSSGLVPRGSHMENLYFQGSWSGGSGGPAELLDEVDSRIDELDSLLASNPTNEELLREILKKILEIFEELFREAKARNDVALLLDAVDLVIDLLDTLLALNPTNEELLREILKIILRIFELLFELAKKLNDVALLLDAVDLVIDLLDTLLALNPTNEELLREILKIILRIFELLFELAKKLNDVALLLDAVDLVIDLLDTLLALNPTNEELLREILKIILRIFELLFELAKKLNDVALLLDAVDLVIDLLDTLLALNPTNEELLREILKIILRIFELLFELAKKLNDTALLSDAIDLVADLLDTLLALNPTNQELLEEIKKIQERIAELEKELLEKLNA
FD14	MGSSHHHHHHSSGLVPRGSHMENLYFQGSWSGGSGGPEELLREVRLRIRELKSLLRSNPTNEELLREILKKILEIFEELFREAKARNDVELLLRAVRLVIRLLKTLLRLNPTNEELLREILKIILRIFELLFELAKKLNDVELLLRAVRLVIRLLKTLLRLNPTNEELLREILKIILRIFELLFELAKKLNDVELLLRAVRLVIRLLKTLLRLNPTNEELLREILKIILRIFELLFELAKKLNDVELLLRAVRLVIRLLKTLLRLNPTNEELLREILKIILRIFELLFELAKKLNDTELLSRAKRLVARLLKTLLRLNPTNQELLEEIKKIQERIAELEKELLEKLNA

FD16*	MGSSHHHHHHSSGLVPRGSHMENLYFQGSWSGGSGGDAEEAAREAEAAIKEAEDEAREAGASEEALKAAKQAFDEIRKAMREAEKSGASEDALEAAAEAFVAIAEAAAEALEAGASEEALKAAAQAFAEIAKAMAEALKSGASEDALEAAAEAFVAIAEAAAEALEAGASEEALKAAAQAFAEIAKAMAEALKSGASEDALEAAAEAFVAIAEAAAEALEAGASEEALKAAAQAFAEIAKAMAEALKSGASEDALEAAAEAFVAIAEAAAEALEAGASEEALKAAAQAFAEIAKAMAEALKSGASEDALEAAAEEFVRIAEAAAEALEAGASEEELREAAKRFAEEAKRMAEELKSGRSA
FD17	MGSSHHHHHHSSGLVPRGSHMENLYFQGSWSGGSGGDADAELREAEEEAKEAAEEAKSDGGDAEAAKKALEFAKKAFDQLREAAKKGADPDAIAELAEAIAEALEAAAEAFSDGGDAEAAAKALEFAAKAFAQLAEAASKGADPDAIAELAEAIAEALEAAAEAFSDGGDAEAAAKALEFAAKAFAQLAEAASKGADPDAIAELAEAIAEALEAAAEAFSDGGDAEAAAKALEFAAKAFAQLAEAASKGADPDAIAELAEAIAEALEAAAEAFSDGGDAEAAAKALEFAAKAFAQLAEAASKGADPDAIASLAEAIAEALERAAETFSDGGDAEEAAKKLEEDAKRFAQKAEEAAKGADP
FD18	MGSSHHHHHHSSGLVPRGSHMENLYFQGSWSGGSGGPEKLASDLEQLAERFEEALARDDEESLRQLLQEFLKIVEKAAKDNNEELLALALELLAELFELALAYNKEELLKLLLQIFLRIVEQAAKAGNEELLALALELLAELFELALAYNKEELLKLLLQIFLRIVEQAAKAGNEELLALALELLAELFELALAYNKEELLKLLLQIFLRIVEQAAKAGNEELLALALELLAELFELALAYNKEELLKLLLQIFLRIVEQAAKAGNEELLALARELLAELQELARAYNKEELLKLLDQIRKRIEDQKRKDGR
FD19	MGSSHHHHHHSSGLVPRGSHMENLYFQGSWSGGSGGQEDLAEDLASTVARAESALEANNNEELRHLSEKLKRLLEELARRGDQELLAELLALIVALAEAALEANNNELLRTLSELLKKLLELLARRGDQELLAELLALIVALAEAALEANNNELLRTLSELLKKLLELLARRGDQELLAELLALIVALAEAALEANNNELLRTLSELLKKLLELLARRGDQELLAELLALIVALAEAALEANNNELLRTLSELLKKLLELLARRGDRELLAELLALISALAEAAQEANNNELKRTREELEKKLSELQSRRGD
FD20	MGSSHHHHHHSSGLVPRGSHMENLYFQGSWSGGSGGREELRQEALRALKDFLKKLEELLARGNREKFAELLASFLEQLEELLAKAFEAGDRELLRQLALEALKIFLKLLELLLARGNRELFAELLALFLALLELLLALAFEAGDRELLRQLALEALKIFLKLLELLLARGNRELFAELLALFLALLELLLALAFEAGDRELLRQLALEALKIFLKLLELLLARGNRELFAELLALFLALLELLLALAFEAGDRELLRQLALEALKIFLKLLELLLARGNRELFAELLALFLALLELLLALAFEAGDRELLRQLAQEARKIFKKLQEELRARGNEELFAELEALFSALEELLDALLQEAGD
FD21	MGSSHHHHHHSSGLVPRGSHMENLYFQGSWSGGSGGREELRQEALRALKDFLKKLEELLARGNREKFAELLASFLEQLEELLAKAFEAGDRELLRQLALEALKIFLKLLELLLARGNRELFAELLALFLELLEELLALAFEAGDRELLRQLALEALKIFLKLLELLLARGNRELFAELLALFLELLEELLALAFEAGDRELLRQLALEALKIFLKLLELLLARGNRELFAELLALFLELLEELLALAFEAGDRELLRQLALEALKIFLKLLELLLARGNRELFAELLALFLELLEELLALAFEAGDRELLRQLAQEARKIFKKLQEELRARGNEELFAELEALFSELEEELDALLQEAGD

FD22	MGSSHHHHHHSSGLVPRGSHMENLYFQGSWSGGSGGREELRQEALRALKDFLKKLEELLARGNREKFAELLASFLEQLEELLAKAFEAGDRELLRQLALEALKIFLKLLELLLARGNRELFAELLALFLALLELLLALAFEAGDRELLRQLALEALKIFLKLLELLLARGNRELFAELLALFLALLELLLALAFEAGDRELLRQLALEALKIFLKLLELLLARGNRELFAELLALFLALLELLLALAFEAGDRELLRQLALEALKIFLKLLELLLARGNRELFAELLALFLALLELLLALAFEAGDRELLRQLAQEARKIFKKLQEELRARGNEELFAELEALFSALEELLDALLQEAGD
FD23*	MGSSHHHHHHSSGLVPRGSHMENLYFQGSWSGGSGGPEEALREVRERIEELKSALESNPTNEEELREILKKILEIFEELFREAKARNDTELLLRAVRAVIELLKTLLELNPTNEELLREILKIILRIFELLFELAKKQNDTELLLRAVRAVIELLKTLLELNPTNEELLREILKIILRIFELLFELAKKQNDTELLLRAVRAVIELLKTLLELNPTNEELLREILKIILRIFELLFELAKKQNDTELLLRAVRAVIELLKTLLELNPTNEELLREILKIILRIFELLFELAKKQNDTELLSRAKEAVAELLRTLAELNPTNQELKEEIKKIQERIAELEKELAEKQNA
FD24	MGSSHHHHHHSSGLVPRGSHMENLYFQGSWSGGSGGPEEALREVRERIEELKSALESNPTNEEELREILKKILEIFEELFREAKARNDVELLLRAVRAVIELLKTLLELNPTNEELLREILKIILRIFELLFELAKKQNDVELLLRAVRAVIELLKTLLELNPTNEELLREILKIILRIFELLFELAKKQNDVELLLRAVRAVIELLKTLLELNPTNEELLREILKIILRIFELLFELAKKQNDVELLLRAVRAVIELLKTLLELNPTNEELLREILKIILRIFELLFELAKKQNDTELLSRAKEAVAELLRTLAELNPTNQELKEEIKKIQERIAELEKELAEKQNA
FD25	MGSSHHHHHHSSGLVPRGSHMENLYFQGSWSGGSGGPEEALREVRERIEELKSALESNPTNEEELREILKKILEIFEELFREAKARNDTELLLRAVRAVIELLKTLLELNPTNEELLREILKIILRIFELLFELAKKLNDTELLLRAVRAVIELLKTLLELNPTNEELLREILKIILRIFELLFELAKKLNDTELLLRAVRAVIELLKTLLELNPTNEELLREILKIILRIFELLFELAKKLNDTELLLRAVRAVIELLKTLLELNPTNEELLREILKIILRIFELLFELAKKLNDTELLSRAKEAVAELLRTLAELNPTNQELKEEIKKIQERIAELEKELAEKLNA
FD26	MGSSHHHHHHSSGLVPRGSHMENLYFQGSWSGGSGGPEEALREVRERIEELKSALESNPTNEEELREILKKIQEIFEELFREAKARNDTELLLRAVRAVIELLKTLLELNPTNEELLREILKIIQRIFELLFELAKKQNDTELLLRAVRAVIELLKTLLELNPTNEELLREILKIIQRIFELLFELAKKQNDTELLLRAVRAVIELLKTLLELNPTNEELLREILKIIQRIFELLFELAKKQNDTELLLRAVRAVIELLKTLLELNPTNEELLREILKIIQRIFELLFELAKKQNDTELLSRAKEAVAELLRTLAELNPTNQELKEEIKKIQERIAELEKELAEKQNA
FD27*	MGSSHHHHHHSSGLVPRGSHMENLYFQGSWSGGSGGPEEALAEVEERIAELESALESNPTNEEELREILKKILEIFEELFREAKARNDTELLLAAVEAVIALLETLLELNPTNEELLREILKIILRIFELLFELAKKQNDTELLLAAVEAVIALLETLLELNPTNEELLREILKIILRIFELLFELAKKQNDTELLLAAVEAVIALLETLLELNPTNEELLREILKIILRIFELLFELAKKQNDTELLLAAVEAVIALLETLLELNPTNEELLREILKIILRIFELLFELAKKQNDTELLSAAKEAVAELLETLAELNPTNQELKEEIKKIQERIAELEKELAEKQNA
FD28*	MGSSHHHHHHSSGLVPRGSHMENLYFQGSWSGGSGGPEEALAEVEERIAELESALESNPTNEEELREILKKILEIFEELFREAKARNDVELLLAAVEAVIALLETLLELNPTNEELLREILKIILRIFELLFELAKKQNDVELLLAAVEAVIALLETLLELNPTNEELLREILKIILRIFELLFELAKKQNDVELLLAAVEAVIALLETLLELNPTNEELLREILKIILRIFELLFELAKKQNDVELLLAAVEAVIALLETLLELNPTNEELLREILKIILRIFELLFELAKKQNDTELLSAAKEAVAELLETLAELN

	PTNQELKEEIKKIQERIAELEKELAEKQNA
FD29	MGSSHHHHHHSSGLVPRGSHMENLYFQGSWSGGSGGPEEALAEVEERIAELESALESNPTNEEELREILKKILEIFEELFREAKARNDTELLLAAVEAVIALLETLLELNPTNEELLREILKIILRIFELLFELAKKLNDTELLLAAVEAVIALLETLLELNPTNEELLREILKIILRIFELLFELAKKLNDTELLLAAVEAVIALLETLLELNPTNEELLREILKIILRIFELLFELAKKLNDTELLLAAVEAVIALLETLLELNPTNEELLREILKIILRIFELLFELAKKLNDTELLSAAKEAVAELLETLAELNPTNQELKEEIKKIQERIAELEKELAEKLNA
FD30	MGSSHHHHHHSSGLVPRGSHMENLYFQGSWSGGSGGPEEALAEVEERIAELESALASNPTNEEELREILKKILEIFEELFREAKARNDVELLLAAVEAVIALLETLLALNPTNEELLREILKIILRIFELLFELAKKQNDVELLLAAVEAVIALLETLLALNPTNEELLREILKIILRIFELLFELAKKQNDVELLLAAVEAVIALLETLLALNPTNEELLREILKIILRIFELLFELAKKQNDVELLLAAVEAVIALLETLLALNPTNEELLREILKIILRIFELLFELAKKQNDTELLSAAKEAVAELLETLAALNPTNQELKEEIKKIQERIAELEKELAEKQNA
FD32	MGSSHHHHHHSSGLVPRGSHMENLYFQGSWSGGSGGPEEALEEVEERIEELESALESNPTNEEELREILKKILEIFEELFREAKARNDVELLLEAVEAVIELLETLLELNPTNEELLREILKIILRIFELLFELAKKQNDVELLLEAVEAVIELLETLLELNPTNEELLREILKIILRIFELLFELAKKQNDVELLLEAVEAVIELLETLLELNPTNEELLREILKIILRIFELLFELAKKQNDVELLLEAVEAVIELLETLLELNPTNEELLREILKIILRIFELLFELAKKQNDTELLSEAKEAVAELLETLAELNPTNQELKEEIKKIQERIAELEKELAEKQNA
FD33	MGSSHHHHHHSSGLVPRGSHMENLYFQGSWSGGSGGPEEALEEVEERIEELESALESNPTNEEELREILKKILEIFEELFREAKARNDTELLLEAVEAVIELLETLLELNPTNEELLREILKIILRIFELLFELAKKLNDTELLLEAVEAVIELLETLLELNPTNEELLREILKIILRIFELLFELAKKLNDTELLLEAVEAVIELLETLLELNPTNEELLREILKIILRIFELLFELAKKLNDTELLLEAVEAVIELLETLLELNPTNEELLREILKIILRIFELLFELAKKLNDTELLSEAKEAVAELLETLAELNPTNQELKEEIKKIQERIAELEKELAEKLNA
FD34	MGSSHHHHHHSSGLVPRGSHMENLYFQGSWSGGSGGPAEALEEVEERIEELESALASNPTNEEELREILKKILEIFEELFREAKARNDTALLLEAVEAVIELLETLLALNPTNEELLREILKIILRIFELLFELAKKQNDTALLLEAVEAVIELLETLLALNPTNEELLREILKIILRIFELLFELAKKQNDTALLLEAVEAVIELLETLLALNPTNEELLREILKIILRIFELLFELAKKQNDTALLLEAVEAVIELLETLLALNPTNEELLREILKIILRIFELLFELAKKQNDTALLSEAKEAVAELLETLAALNPTNQELKEEIKKIQERIAELEKELAEKQNA
FD35	MGSSHHHHHHSSGLVPRGSHMENLYFQGSWSGGSGGKEEALREVRERIEELKRQLESNPHNEEALREILKKILEIFEELFREAKERNDTELLLRAVRAVIELLKTLLELNPHNEELLREILKIILRIFELLFELAKEQNDTELLLRAVRAVIELLKTLLELNPHNEELLREILKIILRIFELLFELAKEQNDTELLLRAVRAVIELLKTLLELNPHNEELLREILKIILRIFELLFELAKEQNDTELLLRAVRAVIELLKTLLELNPHNEELLREILKIILRIFELLFELAKEQNDVELLKRAKEAVDELLRTLAELNPHNEELKEEIKKIEERIEELEKELAEEQND
FD36	MGSSHHHHHHSSGLVPRGSHMENLYFQGSWSGGSGGPEEALAEVEERIAELESALASNPHNEEELREILKKILEIFEELFREAKERNDTELLLAAVEAVIALLETLLALNPHNEELLREILKIILRIFELLFELAKELNDTELLLAAVEAVIALLETLLALNPHNEELLREILKIILRIFELLFELAKELNDTELLLAAVEAVIALLETLLALNPHNEELLREILKIILRIFELLFELAKELNDTELLLAAVEAVIALLETLLALNPHNEELLREILKIILRIFELLFELAKELNDSELLSAAKEAVDALLETLAALN

	PNNEELKEEIKKIEERIEELEKELAEELNS
FD37	MGSSHHHHHHSSGLVPRGSHMENLYFQGSWSGGSGGPEEALEEVEERIEELESALESNPHNEEELREILKKILEIFEELFREAKERNDTELLLEAVEAVIELLETLLELNPHNEELLREILKIILRIFELLFELAKELNDTELLLEAVEAVIELLETLLELNPHNEELLREILKIILRIFELLFELAKELNDTELLLEAVEAVIELLETLLELNPHNEELLREILKIILRIFELLFELAKELNDTELLLEAVEAVIELLETLLELNPHNEELLREILKIILRIFELLFELAKELNDSELLSEAKEAVDELLETLAELNPNNEELKEEIKKIEERIEELEKELAEELNS
FD38	MGSSHHHHHHSSGLVPRGSHMENLYFQGSWSGGSGGPAEALEEVEERIEELESALASNPHNEEELREILKKILEIFEELFREAKERNDTALLLEAVEAVIELLETLLALNPHNEELLREILKIILRIFELLFELAKEQNDTALLLEAVEAVIELLETLLALNPHNEELLREILKIILRIFELLFELAKEQNDTALLLEAVEAVIELLETLLALNPHNEELLREILKIILRIFELLFELAKEQNDTALLLEAVEAVIELLETLLALNPHNEELLREILKIILRIFELLFELAKEQNDSALLSEAKEAVDELLETLAALNPNNEELKEEIKKIEERIEELEKELAEEQNS
FD39	MGSSHHHHHHSSGLVPRGSHMENLYFQGSGGSGGKEKEEALKEILKALEEGGISGELLRQLEEVLKRFLKKAGASEEAILALLEILAALLEGGISIELLLQLLEVLIRFLKKAGLSEEAILALLEILAALLEGGISIELLLQLLEVLIRFLKKAGTSEEEILALLEELAALLEGGISIEELLKRLEEEIKHLKKAGSSW
FD40	MGSSHHHHHHSSGLVPRGSHMENLYFQGSGGSGGPSIEAALKELEEALEAALRELLEAAGVDAEAIERVLEIILRALEEILEIARRAGDDPSFLLALFELAIALLLALFELLVAAGVDAEAIARVLLIILRALIEILEIAVRAGDDPSFLLALFELAIALLLALFELLAAAGVDAEAIARVLLIILRALIEILEIAVRAGDDPSELLALFEAAIARLLALFEALLAQGVDAEAIARRLLEELRKLIERLEEEVRASDEW

Supplementary Table 2: Biochemical and structural properties of the proteins tested in this study.

Name	Size (nm) (l x w x h)	Repeat Spacing (nm)	Net Charge	Number of carboxylate groups	Number of carboxylates at the interface	Ratio of aspartate to glutamate	Solvent accessible surface area (nm^2)	Percent surface hydrophobic
BSA	8.3 x 7.4 x 6.1	n.a.	-18	98	n.a.	39/59	279.33	57.8
FD15	6.4 x 5.5 x 1.8	0.87	-32	67	30	10/57	154.54	51.5
DHR49-Neg	6.7 x 3.7 x 2.3	1.01	-54	72	42	30/42	115.39	46.4
FD31	7.9 x 4.6 x 2.4	1.12	-47	81	36	5/76	150.93	49.3
FD31-Rep3	4.6 x 4.6 x 2.4	1.12	-26	45	18	2/43	89.93	50.1
FD31-Rep9	11.2 x 4.6 x 2.4	1.12	-68	117	54	8/109	220.13	47.4
FD31-Gln-Chkr	7.9 x 4.6 x 2.4	1.12	-29	63	18	5/58	152.12	48.9
FD31-Asp	7.9 x 4.6 x 2.4	1.12	-47	81	36	29/52	148.77	48.7
FD31-Lys-Chkr	7.9 x 4.6 x 2.4	1.12	-23	69	24	5/64	155.38	50.2

Supplementary Discussion

Number density estimation

Given calcite density: 2.7 g/cm^3, MW = 100 g/mol, and assuming all precursor $CaCO_3$ (1 mL 5 mM) is transformed into calcite because of its very low solubility (≈ 1.3 μM), it will produce $CaCO_3$ solids with a total volume of 1.8×10^{17} nm^3. The average cubic-like calcite nanoparticles length ≈ 5.5 nm (Fig. 5b), which gives a volume of ≈ 166 nm^3, enabling us to estimate the number of calcite nanocrystals to be 1.1×10^{15}, which is comparable to the number of protein monomers: 6.5×10^{14} given 1 mL 1.08 μM FD31, and $N_A = 6 \times 10^{23}$.

The electron beam or confinement effects in LP-TEM experiments

To confirm that the observed nucleation of calcite nanocrystals did not result from the continuous exposure to the electron beam or confinement effects, but solely from the interplay between the CO_3^{2-} and the protein-Ca complex, the reaction solution was imaged in a neighboring area without an electron beam irradiation. Calcite nanocrystals with the average size of ≈ 5.5 nm were identified (Fig. 5b). These newly formed calcite nanocrystals keep moving in solution and tend to aggregate, rather than dissolve (Fig. 4, Supplementary Fig. 9). It is plausible that the negatively charged $-COO^-$ in the proteins are absorbed on the calcite nanocrystals, as there are observations of low contrast material on the surface of the nanocrystals (Supplementary Fig. 2c), thus stabilizing them. This is supported by the fact that we failed to observe the gradual growth into bigger rhombohedral calcite crystals as seen in previous *in situ* TEM observations[1]. Further benchtop experiments providing TEM, Cryo-TEM, and *in-situ* liquid-phase ATR-FTIR data confirmed these results for equivalent bulk solutions. We do not expect confinement effects in LP-TEM experiments to affect nucleation rates or pathways since the dimensions for both the calcite nanocrystals (considering a critical nucleus size of ≈ 1-5 nm) and the protein-Ca complex are small compared to those of the liquid-cell.

Proposed mechanisms of protein control over calcite nucleation

Although the precise mechanism of control over nucleation cannot be discerned from the data presented here, given the direct appearance of calcite for certain distributions of carboxylic side chains, the implication is that those distributions bias the configuration of the ions at the protein-solution interface towards that of the calcite lattice. Moreover, because the free energy barrier and critical nucleus size are determined by the probability that ions achieve a size and configuration for which subsequent growth decreases the free energy, this implication is consistent with the rationale originally used for selecting the DHR protein design with its large flat surface and periodic distribution of carboxylic groups.

A variety of factors may explain the different effects of the different proteins. Firstly, the use of odd (Asp) vs. even (Glu) number of carbons in the side chains of the binding moieties could bias the formation of different interfaces, as seen with self-assembled monolayers[2]. Although both Asp and Glu sidechains adopt multiple rotamers, the possible orientations of their carboxyl groups relative to their backbone atoms (and therefore the interactions they can make with a given surface) are distinct. This may contribute to the stabilization of different interfaces by FD31, which exclusively presents Glu and stabilizes calcite {110},

and DHR49-Neg which includes Asp residues and stabilizes calcite {202}. This stereochemical explanation is supported by the observation that mutating Glu residues to Asp ablates the calcite nucleation by FD31, whereas mutating Glu residues to isosteric Gln does not (Fig. 5d-g).

Secondly, geometric lattice matching may play a role. DHRs with different repeat spacings (Supplementary Fig. 3a-c) affect $CaCO_3$ growth differently (Fig. 2), and suggest interactions between specific proteins and specific facets (Fig. 4, Supplementary Fig. 7). Rosetta docking simulations were run with DHR models constrained to be flat and repetitive to identify potential geometric matches at the protein-mineral interfaces (see methods). Since the constrained DHR models with repeat spacings surrounding the minima had comparable predicted energies (Supplementary Fig. 3d), models of FD15, DHR49-Neg, FD31, and FD31 variants (Fig. 5) with repeat distances constrained to ± 1 Å from the minima at 0.1 Å intervals (to represent the possibility of small conformational changes in the proteins) were docked onto models of three calcite facets and one vaterite surface while maintaining repeat symmetry in the protein. The {110} and {202} calcite facets were included based on the observed orientations of nanoparticles formed in the presence of FD31 and DHR49-Neg, respectively, as were the typically expressed calcite {104} and vaterite {010} surfaces. This Rosetta modeling (Supplementary Figs. 13, and 14) did not accurately capture the complex energetics of protein-mineral interactions at solid-liquid interfaces, discriminate between DHRs that nucleate or do not nucleate calcite (Fig. 2), explain the stabilization of specific facets (Fig. 4c, d, Supplementary Fig. 7), or predict the effects of the FD31 surface mutations (Fig. 5d-i). It was consistent with observations that the calcite nucleating DHR proteins bind calcite {110} and {202} rather than the {104} facet (Fig. 4c, d, Supplementary Fig. 7, and 13). Lattice-matching docks were observed both for interfaces that our results suggest template nucleation (e.g., DHR49-Neg on {202} and FD31 on {110}), and those that do not (e.g., FD15 on calcite {110} and FD31 on vaterite {010}; Supplementary Fig. 14). However, these models do demonstrate that DHR proteins are structurally well suited to lattice match $CaCO_3$ surfaces, and to our knowledge the models of DHR49-Neg on calcite {202} and FD31 on calcite {110} are among the best supported models of protein-$CaCO_3$ interfaces that drive heterogeneous nucleation currently available. These models are provided in a GitHub repository alongside the scripts that produced them at https://github.com/fatimadavila/DHR_CaCO3.

Incorporation of proteins into calcite crystals

The results also leave undetermined the extent to which the protein templates are incorporated into the crystals or driven to detach from the nano-calcite surfaces as the crystals assemble. The observation of continuous lattice fringes between attached particles in a number of instances (Fig. 4c and Supplementary Fig. 7h), as well as a significant number of nanocrystals that continue to attach to mature micron-scale calcite crystals (Supplementary Fig. 10e), suggests that at least some proteins do detach and can again act as a template to generate new nano-calcite crystals. In that regard, the protein templates may be thought of as analogous to heterogeneous catalysts: they act as surfaces that reduce the energy barrier to form calcite from $Ca^{2+}$and CO_3^{2-} ions but are not consumed in the process.

Relevance of structured templates to native systems

While numerous native proteins associated with biomineral formation are inherently disordered proteins (IDPs)[3], there are several examples of both structured proteins and proteins that are self-assembled into ordered structures[4,5] or that must interact with a preorganized scaffold[6] before mineralization occurs. Moreover, even in the case of IDPs, whether or not they become structured and/or organized by the time they form the protein-mineral interface is largely unknown.

Supplementary References

1. Nielsen, M. H., Aloni, S. & De Yoreo, J. J. In situ TEM imaging of $CaCO_3$ nucleation reveals coexistence of direct and indirect pathways. *Science* **345**, 1158–1162 (2014).
2. Hu, Q. *et al.* The thermodynamics of calcite nucleation at organic interfaces: Classical vs. non-classical pathways. *Faraday Discuss.* **159**, 509–523 (2012).
3. Boskey, A. L. & Villarreal-Ramirez, E. Intrinsically disordered proteins and biomineralization. *Matrix Biol.* **52-54**, 43–59 (2016).
4. Wang, L. *et al.* Self-assembly and biphasic iron-binding characteristics of Mms6, a bacterial protein that promotes the formation of superparamagnetic magnetite nanoparticles of uniform size and shape. *Biomacromolecules* **13**, 98–105 (2012).
5. Fang, P. A., Conway, J. F., Margolis, H. C., Simmer, J. P. & Beniash, E. Hierarchical self-assembly of amelogenin and the regulation of biomineralization at the nanoscale. *Proc. Natl. Acad. Sci. U. S. A.* **108**, 14097–14102 (2011).
6. Gal, A. *et al.* Macromolecular recognition directs calcium ions to coccolith mineralization sites. *Science* **353**, 590–593 (2016).
