# Supplementary material for: Directing polymorph specific calcium carbonate formation with de novo protein templates
Source: Nat Commun. 2023 Dec 14;14:8191. doi: 10.1038/s41467-023-43608-1 (PMC10721895; doi:10.1038/s41467-023-43608-1)
Supplement: Supplementary file 3 — Description of Additional Supplementary Files [file 41467_2023_43608_MOESM3_ESM.pdf]

Title: Supplementary Movie 1:
Description: LP-TEM showing the nucleation of calcite nanocrystals in solution.

Title: Supplementary Movie 2:
Description: LP-TEM showing the nucleation of calcite nanocrystals at the protein-Ca^{2+} assemblies.

Title: Supplementary Movie 3:
Description: LP-TEM showing the particle attachment mediated growth of calcite nanocrystals.
